# A pilot study of the use of near-patient C-Reactive Protein testing in the treatment of adult respiratory tract infections in one Irish general practice

**DOI:** 10.1186/1471-2296-12-93

**Published:** 2011-08-31

**Authors:** Kim E Kavanagh, Eamonn O'Shea, Rita Halloran, Peter Cantillon, Andrew W Murphy

**Affiliations:** 1 Discipline of General Practice, National University of Ireland Galway, 1 Distillery Rd, Newcastle, Galway, Ireland; 2 The Claddagh Medical Centre, 4 The Crescent, Galway, Ireland; 3 Irish College of General Practitioners, 4/5 Lincoln Place, Dublin 2, Ireland

## Abstract

**Background:**

New approaches are being sought to safely reduce community antibiotic prescribing. A recent study demonstrated that CRP testing resulted in decreased antibiotic prescribing for lower respiratory tract infection in primary care. There is little other published primary care data available evaluating CRP in the treatment of lower respiratory tract infections in routine clinical practice. This pilot study aims to describe the performance of near-patient CRP testing, in a mixed payments health system. Specific areas to be reviewed included the integrity of the study protocol, testing of data collection forma and acceptability of the intervention.

**Patients**

Patients over the age of 18 years, with acute cough and/or sore throat with a duration of one month or less, in routine clinical practice.

**Method:**

Design: A pilot with a cross-sectional design. The first 60 recruited patients were treated with routine clinical management, and GP's had no access to a CRP test. For the subsequent 60 patients, access to CRP testing was available.

Participants: 3 GP's in one Irish primary care practice recruited 120 patients, fulfilling the above criteria over five months, from January 1 to May 31, 2010.

Main outcome measures: The primary outcome was antibiotic prescription at the index consultation. Secondary outcomes were the numbers of delayed prescriptions issued, patient satisfaction immediately after consultation and re-consultations and antibiotic prescriptions during 28 days follow-up.

**Results:**

The protocol and data collection forms worked well and the intervention of CRP testing appeared acceptable. Thirty-five (58%) patients in the no-test group received antibiotic prescriptions compared to 27 (45%) in the test group. Both groups demonstrated similarly high level of patient satisfaction (85%). Fourteen (23%) patients in the CRP test group re-attended within 28 days compared to 9 (15%) in the no-CRP test group.

**Conclusion:**

This pilot study confirms the potential feasibility of a full trial in Irish general practice. Further consideration of possible increased re-attendance rates in a mixed payments health system is appropriate. We intend to pursue a larger trial in a newly established regional primary care research network.

## Background

Acute respiratory tract infections are extremely common in general practice and are the commonest reason for general practitioner consultation [1]. Most of these infections are self-limiting with limited if any benefit from antibiotic use [2]. Excessive antibiotic use has significant implications including cost, medicalisation of self-limiting illness, increased potential for adverse effects and development of resistance [2]. New approaches to reduce antibiotic prescribing include patient and doctor education, utilizing delayed antibiotic prescribing and near-patient/point of care CRP testing [3].

CRP testing is emerging as an important aspect of routine general practice care and several point of care devices now exist [3]. The CRP assay is used widely in Nordic countries. In Norway a CRP test is now performed in one out of every 8 consultations and a study in Sweden in 2002 revealed that a CRP test was carried out in 41% of all patients consulting a GP with an airway infection [4].

A recent systematic review addressing the diagnostic value of CRP in discriminating between bacterial and viral infections of the lower respiratory tract [4] called for further research given the poor methodological quality of the studies included, and commented that the current available evidence was not consistently sufficient in supporting the use of CRP to guide antibiotic prescribing in respiratory tract infections.

Of further interest, a subsequent pragmatic trial published in the BMJ in 2009 studied general practitioners' use of C reactive protein testing and training in communication skills in patients with acute cough. This demonstrated that both methods resulted in decreased antibiotic prescribing for lower respiratory tract infection in primary care. Patient recovery and satisfaction with care were not compromised and the two approaches combined resulted in the greatest reduction in antibiotic prescribing [5]. Other research has suggested that near patient CRP testing in general practice could lead to a reduction in antibiotic prescribing in patients with sinusitis [6].

Like most other European countries [7], the majority of patients in the Republic of Ireland make significant out of pocket contributions for primary care. In the Republic of Ireland, free healthcare and medications are available to patients who qualify under the means-tested General Medical Scheme (GMS). GMS patients account for approximately 29% of the population [8] and therefore represent the least affluent sector of society. The remainder, referred to as 'Private' patients, whose income is above a certain level, are required to pay for all of their own healthcare costs. The necessity for the majority of the population to pay the out of pocket costs for GP care in full (typically € 50 per consultation), adds another significant consumer dimension to the implementation of CRP testing in Ireland namely the impact of patient satisfaction and cost on consultations and outcomes. Therefore, we felt there was a need for a pilot study in an Irish setting to determine the feasibility of near patient CRP testing in routine clinical practice on the treatment of respiratory tract infections. As suggested by Lancaster (2004) [9] and Arain (2010) [10], specific areas to be reviewed included the integrity of the study protocol, testing of data collection forma and acceptability of the intervention.

## Method

This was a pilot of 120 patients with a cross-sectional design. Patients were recruited over a five-month period from January to May 2011. The first 60 recruited patients were treated with routine clinical management, and GP's had no access to a CRP test. For the subsequent 60 patients, access to CRP testing was available. Each patient completed two initial questions referring to perceived need for, and expectation of, antibiotic treatment once it was established by the doctor that their presenting symptoms met inclusion criteria for the study, prior to examination by the doctor. Patients were advised to complete the final question in relation to their level of satisfaction with the consultation on a Likert scale, when the consultation was complete and once they had left the consulting room. They were then asked to leave their completed questionnaire in a sealed box at the practice reception area. During the same consultation, while the patient was present, the doctor also completed a similar questionnaire regarding the working diagnosis and two further questions relating to their perception of the patient's expectations in relation to antibiotic prescription, and their opinion in relation to clinical indication for antibiotic prescription (after examination but prior to CRP testing if in that arm of the study).

At the end of the study period, all three doctors met in an unstructured format to discuss their reflections on the study, in particular with reference to perceived advantages, disadvantages and potential areas for future use of the CRP test. Identified themes were recorded in writing by KK,

Setting: The study was carried out in one suburban general practice in Ireland catering for 6200 patients, (both GMS and private), by 3 of the 6 general practitioners (Dr's A, B and C), 2 of whom were employed on a full time basis and one part- time.

Participants and consent: All adult patients aged 18 years or older with acute cough and/or sore throat with duration of one month or less were recruited. A consecutive sampling method was used by the physicians involved in the study to select eligible patients in the course of routine practice. Information posters were placed in all practice waiting rooms explaining that patients may be asked to complete a questionnaire and/or have a blood test if they presented to a physician with a cough and/or a sore throat. When a patient meeting the inclusion criteria presented to one of the physicians involved (generally apparent once the presenting complaint was elicited by the physician), the patient was then asked if they would like to take part in the study. A verbal description of the study was given at the beginning of each relevant consultation, and the voluntary nature of participation was emphasized at all stages. Following full verbal description of the study, informed consent was obtained verbally from each eligible patient. For repeat attendances, clinical management including whether to perform repeat CRP testing, was entirely at the discretion of the GP involved,

Ethical approval was granted, by the Irish College of General Practitioners Ethics Committee.

### Variables

Primary outcome was antibiotic prescription at the index consultation.

Secondary outcomes were number of delayed prescriptions issued, re-consultation (referring to both 'in person' and telephone consultations) and antibiotic prescription, both during 28 days of follow-up, and patient satisfaction. Repeat attendances within 28 days and antibiotic prescription at re-attendance were recorded but not included as new patient encounters in analysis.

Data sources/Measurement: CRP measurements were performed using the point of care Quik-Read^® ^CRP kit, Orion Diagnostica Oy [11]. This device has been validated in a previous study and has shown good concordance with standard laboratory CRP measurement [12]. There is a general agreement that the normal range of CRP at all ages is 0-10 mg/l [13]. For the purposes of our study, a CRP value of less than 20 was considered indicative of a viral or self-limiting infection. A value of 20-50 was taken to indicate a 'borderline' level, (at which advice would usually be given to observe symptoms over 48 hrs with explanation in relation to red flag symptoms and signs, and the possible issue of a delayed antibiotic prescription). A level of > 50 was considered to be indicative of a bacterial infection [14].

Upon completion of the study period, outcome data was extracted by one of the study coordinators (KK). As suggested by Lancaster [9] and Arain [10], formal statistical analysis of pilot results was not performed.

## Results

### Participants

One hundred and twenty participants were recruited in total, sixty to each arm of the study over a 5-month period from January to May 2010. All (100%) of patients recruited in both arms agreed to be involved in the study. One patient in the 'CRP arm' refused a blood test due to needle phobia; however this patient still agreed to complete a questionnaire.

### Descriptive data

The mean ages of participants were similar in both 'CRP' and 'No CRP' arms, at 47.6 years (SD 16.3) and 48 years (SD 17.8) respectively. Forty eight percent of patients in the 'No CRP' group were private, and 52% were GMS compared to 37% private and 63% GMS in the 'CRP' group. In the 'No CRP' group, patient questionnaires were missing for 3 of the 60 participants (5%) and doctor's questionnaire was missing for 1 participant (1.7%). In the 'CRP' group, patient questionnaires were missing for 3 of the 60 participants (5%) and there were no missing doctor's questionnaires. One patient in the 'CRP' group refused the CRP testing due to a needle phobia.

Both groups were broadly similar in terms of working diagnosis. Thirty-eight patients (63%) in each group were diagnosed with Upper Respiratory Tract Infections with 19 (32%) and 16 (27%) diagnosed with Lower Respiratory tract infections in the 'No CRP' and 'CRP' groups respectively. (The remainder of patients in each group, were diagnosed with a respiratory tract infection whose site was not specified, or had a non- infectious diagnosis (e.g rhinitis). The consecutive opportunistic sampling method used meant that participants were not distributed uniformly between the three physicians (denoted by letters below) with the breakdown as follows: Dr A 32% and 22% of participants in the 'no CRP' and 'CRP' groups respectively, Dr B 38% and 50% respectively, and Dr C 28% and 43% respectively.

### Outcome data

Table 1 outlines the results of the outcomes considered in this study, comparing the initial 'no CRP' arm, where no CRP assays were performed, and the 'CRP' arm where CRP assays were performed on every patient. There was a reduction in antibiotic prescribing in the 'CRP' arm (45% v 58.3%). A higher proportion of those who received an antibiotic prescription in the 'CRP' group received a delayed antibiotic prescription (37% v 28%). There was also an increase in re-consultation rates in the 'CRP' group with 25% re-presenting with a similar problem within 1 month, compared to 15% in the 'No CRP' group. Both telephone and in-person consultations were included in the re-consultation figures. The majority of patients who re-consulted in each group had a GMS card: 56% in the 'No CRP' group versus 78% in the 'CRP' group.

**Table 1:** Outcome data

	No CRP arm		CRP arm	
	%	n	%	n
Antibiotic prescribed at index consultation	58.3	35	45	27
Immediate antibiotic prescription	71.4	25	63	17
Delayed antibiotic prescriptions	28	10	37	10
Patient's expectation of antibiotic prescription:				
Yes	48.3	29	36.6	22
No	8.3	5	11.7	7
Unsure	40	24	46.7	28
Doctor's perception of patient's expectation for antibiotic prescription				
Yes	55	33	35	21
No	8.3	5	10	6
Unsure	35	21	55	33
Doctor's opinion on necessity for antibiotics:				
Yes	25	15	18.3	11
No	53.3	32	80	48
Unsure	18.3	11	1.7	1
Re-consult within 28/7 with similar problem	15	9	23	14
% of re-consults who received antibiotic prescription at re-presentation	33.3	3	46.7	7
Patient Satisfaction (Satisfied and Very satisfied)	85	51	85	51

This table depicts the results of the outcomes considered in this study, comparing the initial 'no CRP' arm, where no CRP assays were performed, and the 'CRP' arm where CRP assays were performed on every patient.

Seventy eight percent (n = 7 out of 9) of those who re-consulted in the 'no CRP' group had received an antibiotic prescription in their first consultation, compared to 57% (n = 8 out of 14) in the 'CRP' group.

Of those who re-consulted in the 'No CRP group', one third received an antibiotic prescription, compared to almost one half of the cohort who re-consulted in the 'CRP' group.

Patients' expectation of receiving an antibiotic prescription was lower in the 'CRP' group (36.6% v 48.3%). There was also a decrease in doctor's level of uncertainty with regard to antibiotic necessity in the CRP group with only 1.7% stating they were uncertain as to whether the patient required an antibiotic after examination compared to 18.3% of the 'No CRP' group.

Doctors' perception of patient expectation for antibiotic prescription differed to patients' self professed expectation for antibiotic prescription between the two groups at 55% v 48.3% in the 'No CRP' group and 35% v 36.6% in the 'CRP' group..

Patient satisfaction was similarly high in both groups with 85% reporting they were 'Satisfied' or 'Very satisfied' with the consultation.

Table 2 outlines the CRP values and antibiotic prescription rates (both immediate and delayed) for patients in the 'CRP' arm of study. The lowest proportion of patients (8%) had a CRP level > 50 and there was a 100% antibiotic prescription rate in this group, with no delayed prescriptions. Approximately half of all patients fell into the 'borderline' CRP (20-50) group with an antibiotic prescription rate of 56.6%, and just over half of these (53%) were delayed prescriptions, representing the highest rate of delayed prescriptions in these 3 subgroups. Two fifths of patients had a CRP level of < 20, with an antibiotic prescribing rate of 20% in this subset. As mentioned above, one patient refused CRP testing due to needle phobia.

**Table 2:** 'CRP values and antibiotic prescription in 'CRP' arm of study'

CRP Value		Immediate Antibiotic prescriptions	Delayed antibiotic prescriptions
	n	n	N
< 20	24 (40%)	4	1
20-50	30 (50%)	8	9
> 50	5 (8%)	5	0

This table demonstrates the CRP values and number of immediate and delayed antibiotic prescriptions among patients in the CRP arm of the study. In the table patients are divided into 3 groups according to CRP level. (Note-1 patient refused a blood test. Percentages are calculated out of 60-the total patient number in this group.)

### Practitioner reflections

All practitioners agreed that they felt the use of CRP testing was beneficial and had increased their confidence to withhold antibiotic prescriptions in certain patients (for example asthmatics, and patients with cough with no localizing signs who felt themselves that they should get an antibiotic prescription). It was highlighted that performing this test did add cost (to the GP in terms of test materials used) and time to the consultation. This time reduced with experience but was estimated at up to 3 minutes in some consultations. It was postulated however, that this extra, invested time may have been a trade-off improving patient education and changing future behaviour in some cases It was suggested that the utility of CRP testing may be its role as a rule-in tool in pivotal patients such as those above, and/or as a bargaining tool in those consultations where there was some conflict or uncertainty for example where the patient demanded antibiotics but in whom they were not felt to be clinically necessary.

## Discussion

The protocol and data collection forms worked well and the intervention of CRP testing appeared acceptable. Preliminary findings suggest that the use of near-patient CRP testing may be associated with reduced antibiotic prescribing for respiratory tract infections, high levels of patient satisfaction and increased re-consultations.

### Implications of pilot

We feel that this pilot study confirms the potential feasibility of a full trial in Irish general practice and reveals two important lessons. First, it was originally intended to spend 6 weeks recruiting patients for each study arm, commencing in January 2010 thus completing data collection at the end of March 2010, however this time frame was extended as recruitment for the initial arm proved slower than expected due to an unexpected reduction in patient attendances in January 2010. This is in contrast to current (at time of writing) attendances in the midst of an influenza epidemic, which could potentially be an ideal time to undertake a future study such as this. Second, the provision of a denominator (i.e documentation of the total number of eligible patients who presented during the study period) in a future, more extensive study would enhance understanding of the generalisability of study results [15].

Other important learning points, based on analysis of study results, along with qualitative reflections of practitioners involved were as follows:

### Re-consultation rates

We noted an increase in re-consultation rates within one month of the index consultation in those patients in the "CRP' arm. Of note, a higher proportion of those who re-consulted in the 'no CRP' group had received an antibiotic compared to those who re-consulted in the 'CRP' group (78% v 57%). When the baseline characteristics of both groups are examined, a larger proportion (63% v 52%)of the 'CRP' group had GMS cards (ie did not pay for consultations), which may have had an influence on likelihood of re-consultation. A previous large study, looking at the effect of CRP testing and or communication skills on antibiotic use in lower respiratory tract infections, found a slight increase in re-consultation rates using CRP measurement, however this increase was not statistically significant [5].

Given the small numbers in this study it is difficult to draw accurate conclusions from the above figures, A future larger study would need to address the above trend and seek further definition of the re-consulting population, including qualitative information on reasons for re-consultation.

### Practice Resources

One factor highlighted in practitioners' reflections, which may limit the use of this test in routine practice, is the financial cost of test materials and reagent, which many practices could find prohibitive.

### Patient satisfaction

We showed that the use of CRP in the management of respiratory tract infections in our practice did not cause a reduction in patient satisfaction. We demonstrated a similarly high level of patient satisfaction in both study groups and rates of satisfaction were similar in both GMS and private patients. Only one patient in the study refused CRP testing and this was due to needle phobia.

### Practitioners' perspectives

In general many of the sentiments expressed by the GP's involved in this study echo previously published qualitative research themes in this area [16]

### Limitations

Two major limitations in this study are the issues of size and study duration. This study was conducted in 120 patients in one general practice during only one winter/spring season. Further studies would need to involve a larger population over a longer period of time to confirm or refute any trends we noted. In a future study seasonal factors would be taken into account.

Although all study practitioners were in agreement in terms of criteria for patient eligibility and selection, future studies may want to refine the consecutive approach we took to sampling in the course of routine practice, to reduce any possibility of 'cherry picking'.

We included patients with both upper and lower respiratory tract infections in this study. Much previous work on the use of CRP levels has been in reference to its use in lower respiratory tract infections. In addition we did not document illness duration prior to presentation as a study outcome. Both of the above factors may have had an influence on interpretation of CRP values. Previous research has suggested that raised CRP in viral infections during the first week can mislead clinicians into prescribing antibiotics, and have suggested that moderately elevated CRP values in patients with respiratory tract infection cannot support a diagnosis of bacterial infection when the illness has lasted less than 7 days [17]. Further and potentially larger studies may benefit from analyzing results of upper and lower respiratory tract infections separately and making note of illness duration.

## Conclusions

This pilot study confirms the potential feasibility of a full trial in Irish general practice. It re-iterates that the use of near-patient CRP testing may be associated with reduced antibiotic prescribing for respiratory tract infections and suggests it may be associated with high levels of patient satisfaction and increased re-consultations. With the establishment of the Western Research and Education Network (WestREN), a new primary care research network in the West of Ireland [18] we now have the potential to conduct a subsequent larger study through up to 80 general practices covering over 200,000 patients.

## Competing interests

The Discipline of General Practice, NUI Galway has received unrestricted educational funding from MSD, Menarini and Pfizer pharmaceutical companies. This funding has been solely used to support educational meetings for general practitioners who take medical students from NUI Galway. The Claddagh Medical Centre is a member of WestREN [18] and received an unrestricted WestREN Research Bursary funded by MSD to support conduction of this study.

## Authors' contributions

KK participated in design of the study, was one of the 3 General Practitioners who selected patients and distributed questionnaires, analysed all data, drafted the manuscript and is corresponding author. EOS and RH are principal General Practitioners in the practice where the study was conducted, were responsible for conception of the original study idea, designed the original questionnaires used in this study and were the remaining 2 GP's who selected patients and distributed questionnaires. PC and AWM participated in study design and supervised data analysis and manuscript redrafting throughout. All authors critically reviewed the manuscript and gave their final approval of the version of the manuscript submitted for publication.

## Pre-publication history

The pre-publication history for this paper can be accessed here:

http://www.biomedcentral.com/1471-2296/12/93/prepub
